# Picosecond pulse generation from continuous-wave light in an integrated nonlinear Bragg grating

**DOI:** 10.1515/nanoph-2022-0026

**Published:** 2022-03-18

**Authors:** Ju Won Choi, Byoung-Uk Sohn, Ezgi Sahin, George F. R. Chen, Doris K. T. Ng, Benjamin J. Eggleton, Carel Martijn de Sterke, Dawn T. H. Tan

**Affiliations:** Photonics Devices and System Group, Singapore University of Technology and Design, 8 Somapah Rd, Singapore 487372, Singapore; Institute of Microelectronics, A*STAR, 2 Fusionopolis Way, #08-02, Innovis Tower, Singapore 138634, Singapore; Institute of Photonics and Optical Science, School of Physics, The University of Sydney, Sydney, NSW 2006, Australia; The University of Sydney Nano Institute (Sydney Nano), The University of Sydney, Sydney, NSW 2006, Australia

**Keywords:** CMOS-compatible devices, nonlinear Bragg gratings, nonlinear optics, pulse train generation, ultra-silicon-rich-nitride

## Abstract

The generation of optical pulse trains from continuous-wave light has attracted growing attention in recent years because it provides a simple way to obtain high repetition rate ultrashort pulses. While pulse generation has been extensively demonstrated in optical fibers, pulse train generation from weak, continuous wave light in photonic chips has posed significant challenges because of the short interaction length and therefore difficulty in acquiring sufficient new frequency content, and/or absence of the appropriate dispersion environment. In this manuscript, we report the pulse train generation of a low continuous-wave signal to 18 ps, by leveraging cross-phase modulation induced by co-propagating pump pulses with a peak power of 3.7 W in an ultra-silicon-rich nitride grating. The pulse train generation dynamics are documented both experimentally and theoretically to arise from cross-phase modulation-induced generation of new spectral content, and dispersive re-phasing. This is a new approach in which picosecond pulse generation may be achieved from low power, continuous-wave light.

## 1 Introduction

Pulse train generation from continuous-wave (CW) light has extensively demonstrated using a variety of different approaches in optical fibers. Their theoretical foundations were first formulated in the 1980s when quasi-periodic solutions to the nonlinear Schrödinger equation were found to exist in the presence of anomalous dispersion [1]. Hasegawa reported that CW light propagating in the anomalous dispersion regime of an optical fiber could generate a picosecond pulse train induced by modulation instability [2]. These predictions were extended to modulational polarization instabilities which showed that birefringent optical fiber could likewise create pulse trains regardless of the fiber dispersion [3], as well as implementation in cavities in order to reduce the high powers required for the onset of modulation instability in optical fibers [4]. Different paradigms of pulse train generation involving cross-phase modulation (XPM) and four-wave mixing were developed after Hasegawa’s scheme was introduced. In the former, XPM was used to trigger modulation instability [5], or generate frequency chirp in a CW signal propagating in media with negative dispersion [6]. The latter is a comparatively newer method, relying on multiple four-wave mixing (FWM) phenomena in optical fiber [7, 8]. Over the years, experimental advances in generating pulse trains have been made in various media including fiber ring cavities, highly nonlinear fiber and fiber Bragg gratings [9], [10], [11], [12].

While significant progress has been made in these pulse train generation schemes, on-chip pulse train generation using CW light has not been demonstrated. To achieve this, several challenges need to be overcome, including the smaller effective path lengths available in waveguides which could result in a smaller overall nonlinear effects. This may be offset to some degree by the much larger nonlinearity available in certain integrated platforms which may have nonlinear parameters six orders of magnitude larger than in optical fiber [13], [14], [15]. In addition, existing modulation instability-based pulse train generation in optical fiber could encounter competition from stimulated Brillouin scattering, which could cause modulation instability and therefore the pulse train generation process to deteriorate. Conversely, the pulse generation schemes demonstrated in fiber relying on XPM also face difficulties in on-chip implementation. The signal undergoing XPM needs to experience a sufficient magnitude of dispersion to acquire a pulsed nature. This is difficult to achieve in on-chip waveguides where it is typical for dispersive lengths to far exceed the nonlinear lengths [13], [14], [15], [16].

In this paper, we report the first demonstration pulse generation from CW-light on a chip, using an integrated Bragg grating. This chip-scale pulse train generation scheme is distinct from the aforementioned techniques which rely on modulation instability. The pulse dynamics are underpinned by XPM of a weak CW signal by a co-propagating pump. We experimentally observe XPM of a pump inducing a bidirectional squeezing effect onto a weak probe. Under the right conditions, this results in picosecond pulse generation from low power CW light. We demonstrate the generation of 18 ps pulse trains from low-power (1 mW) CW light, using co-propagating pump pulses at a low peak power of 3.7 W. Strong anomalous dispersion induced by an on-chip grating is both experimentally and theoretically confirmed to play a central role in the signal waveform’s evolution. Experimental agreement with numerical calculations allows us to describe the dynamics underpinning the observed pulse train generation.

## 2 Picosecond pulse generation from continuous-wave light

**Figure 1:**
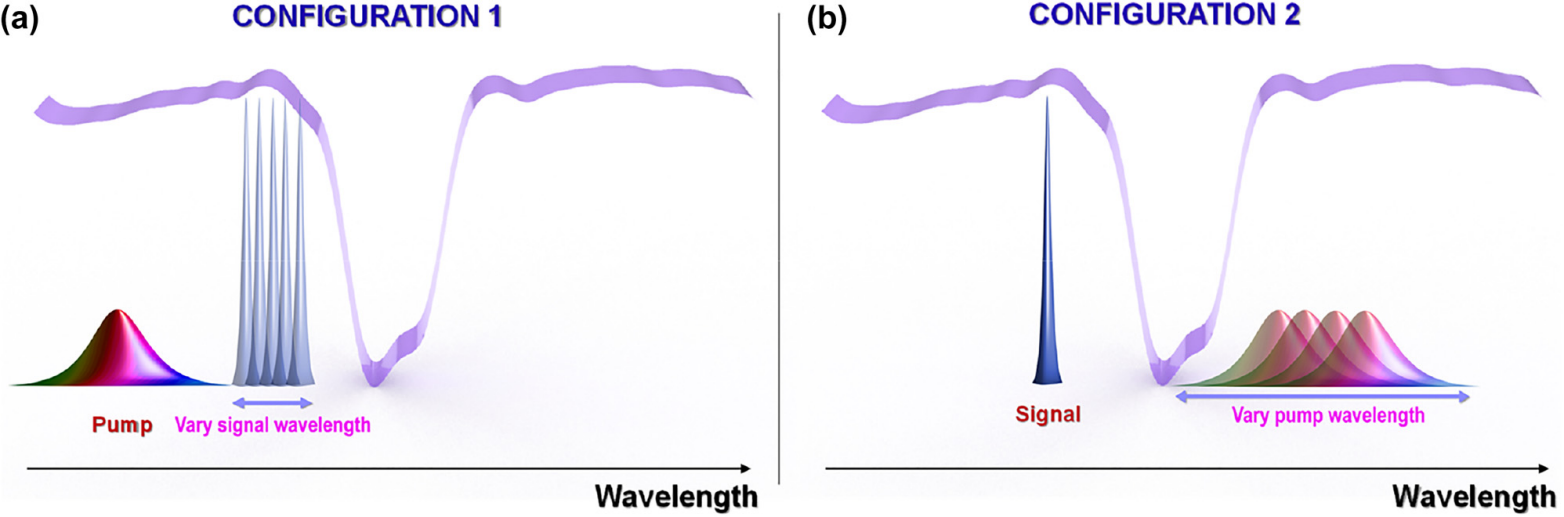
Two pump-probe configurations for achieving XPM-based pulse train generation, relative to the grating stopband (purple). (a) Configuration 1: signal wavelength is tuned close to the blue edge and pump wavelength is fixed far from the grating resonance. (b) Configuration 2: pump wavelength is tuned close to the red edge and signal wavelength is fixed on the blue edge. The grating spectrum, pump and signal placement are drawn versus wavelength.

Picosecond pulse train generation from a CW signal is studied in the context of a nonlinear grating [17], [18], [19] implemented on an ultra-silicon-rich nitride (USRN) chip [20], [21], [22], [23]. Figure 1 illustrates the operating principles. Central to the generation of picosecond pulses from CW light studied in this paper, is the large, grating-induced anomalous dispersion on the blue side of the stopband, and the grating nonlinearity, which benefits from a similar slow light scaling also found in photonic crystal waveguides [21, 24]. The magnitude of the band-edge dispersion was previously reported to be three orders of magnitude larger than in conventional waveguides [17]. We first describe Configuration 1 in which the pump is located far from the grating stopband, and its dispersion is dominated by the background waveguide dispersion, which is negligible over the length scale of the experiment. In contrast, the signal is placed close to the blue side of the stopband where the group velocity of an incident optical field decreases rapidly as its wavelength increases. Through XPM, the signal acquires both blue- and red-shifted frequencies: The leading (trailing) edge is red- (blue-) shifted. The grating provides strong anomalous dispersion, such that red- (blue-) shifted wavelengths are temporally retarded (accelerated). Consequently, the effect of the pump is two-fold. Part of the probe’s energy experiences a heaping effect inwards from the leading edge of the pump. Yet the other part of the probe’s energy experiences a heaping effect, also inwards, from the trailing edge of the pump. The combined effect is to cause the CW probe to squeeze or pull itself inwards as it interacts with the pump. Two important points can be made here: (i) the CW probe experiences an inward-pulling effect as a result of a co-propagating pump, whereas (ii) this pulling effect occurs at both sides of the portion of the CW probe which overlaps with the pump.

**Figure 2:**
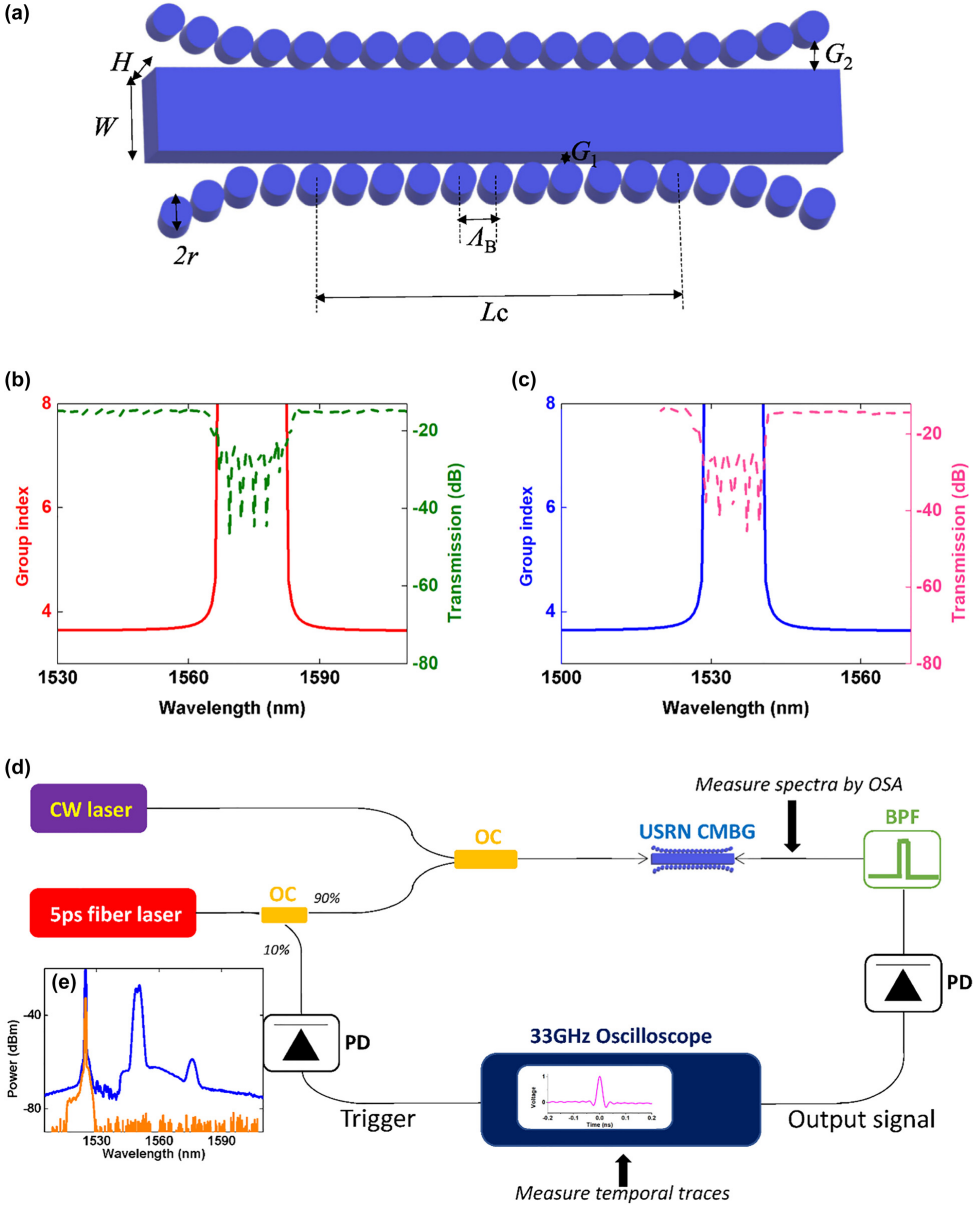
(a) The schematic the grating used and linear transmission spectra and group index properties for the nonlinear gratings used in (b) Configuration 1 and (c) Configuration 2. (d) The experimental setup for temporal and spectral measurements. (OC: optical coupler, OSA: optical spectrum analyzer, BPF: bandpass filter, PD: photodetector). (e) The output spectra before BPF (blue line) and after BPF (orange line) for Configuration 2.

In our experiments, we utilize a grating with a stopband centered at 1575 nm (3 dB bandwidth = 15 nm) with a length of 3 mm (schematic shown in Figure 2a). The gratings (Figure 2a) are fabricated on the complementary metal-oxide semiconductor compatible USRN platform with a linear and nonlinear refractive index of 3.1 and 2.8 × 10^−13^ cm^2^/W at 1550 nm, respectively. USRN does not exhibit two-photon absorption at telecommunications wavelengths [23]. The nonlinear grating is based on a cladding-modulated Bragg grating (CMBG) as shown in Figure 2a. The nonlinear grating induces a periodic modulation of the effective index using periodic pillars placed at fixed distance *G*_1_ from a central waveguide. The total grating length (*L*) is 3 mm with a 200 μm apodized section on each end of the grating. The central length (*L*_c_), possessing a constant distance between pillar and central waveguide is 2.6 mm. The pillar diameter (2*r*) is 200 nm. The gap distance between the central waveguide and pillar is 50 and 150 nm at center (*G*_1_) and ends (*G*_2_), respectively. Apodization lengths (200 μm each) implemented at both ends of the grating are necessary to reduce both transmission and phase ripple. The central waveguide has a width (*W*) of 600 nm and height (*H*) of 320 nm. Figure 2b and c show the experimentally measured transmission and group index properties of the gratings used in Configurations 1 and 2 (to be discussed below), respectively. The stopband of the nonlinear gratings used in Configurations 1 and 2 is centered at 1575 and 1535 nm respectively, with a 3 dB bandwidth of 15 nm. The group indices were measured using a dispersion analyzer, whereas the transmission spectrum of the gratings was measured using an amplified spontaneous emission source and an optical spectrum analyzer. When the wavelength is far from the band edges of the photonic bandgap, the group indices are unaltered by the grating and possess the same value as in a USRN photonic waveguide without the grating. We note that the transmission spectrum of the grating shown in Figure 2c terminates at 1520 nm because this is the lower wavelength limit of the ASE source used to measure the transmission spectrum. However, we note that the overall transmission spectrum of this grating should take a similar form as that in Figure 2b, as the two gratings have the same design but with a different pitch to target different center wavelengths. In addition, we note that the group index of the grating varies rapidly close to the grating stopband. Far from the stopband, the group index approaches the same value as a waveguide of the same dimensions. This is corroborated by the measurements of the group index in Figures 2b and c, which show negligible change in the group index far from the grating stopband. Within the grating stopband, light slows significantly and undergoes reflection. We note further that the region within the stopband was not used in our work.

Experimental characterization of the CW pulse train generation dynamics is performed using the experimental setup as shown in Figure 2d. 5 ps pulses from a fiber laser delivering hyperbolic secant pulses with a pump power of *P*_0,pump_ = 3.7 W, are used as the pump (repetition rate = 20 MHz, wavelength = 1550 nm). The pump pulses possess a 3 dB bandwidth of 0.6 nm, possessing a time-bandwidth product of ∼0.37, indicating that the pump pulses are nearly transform-limited. The pulse wavelength is tunable from 1540–1560 nm. The fiber laser incorporates a thin film with a bandpass coating on one side and an anti-reflection coating on the other side. The wavelength tunability is achieved by rotating the thin film such that the angle of incidence may be varied. Decreasing the angle of incidence reduces the pulse wavelength.

The pump pulses are combined with the CW signal with a power of *P*_signal_ = 1 mW using 3 dB coupler and coupled into the CMBG via tapered fiber. The output spectra were measured with an optical spectrum analyzer. To measure temporal traces of amplified signals, the bandpass filter with high suppression of noise level after output FWM spectrum was used for filtering out amplified signal. The blue line in Figure 2e shows the spectrum before the signal is carved out for measurement when using Configuration 2. As we wanted to characterize the pulse generation occurring in the CW signal when undergoing the combined effect of XPM and group velocity dispersion (GVD), we filtered out the pump and idler while retaining only the signal. Both pump and idler are removed using a band pass filter. The orange line in Figure 2e illustrates the signal that is temporally characterized, where the pump and idler have been filtered out using the band pass filter. The filtered signals undergo optical-electrical conversion using a photodetector with 33 GHz bandwidth, prior to the measurement of the temporal traces using an oscilloscope with a bandwidth of 33 GHz. 10% of the power from the 5 ps pulses was routed to a 5 GHz photodetector to trigger the oscilloscope traces.

**Figure 3:**
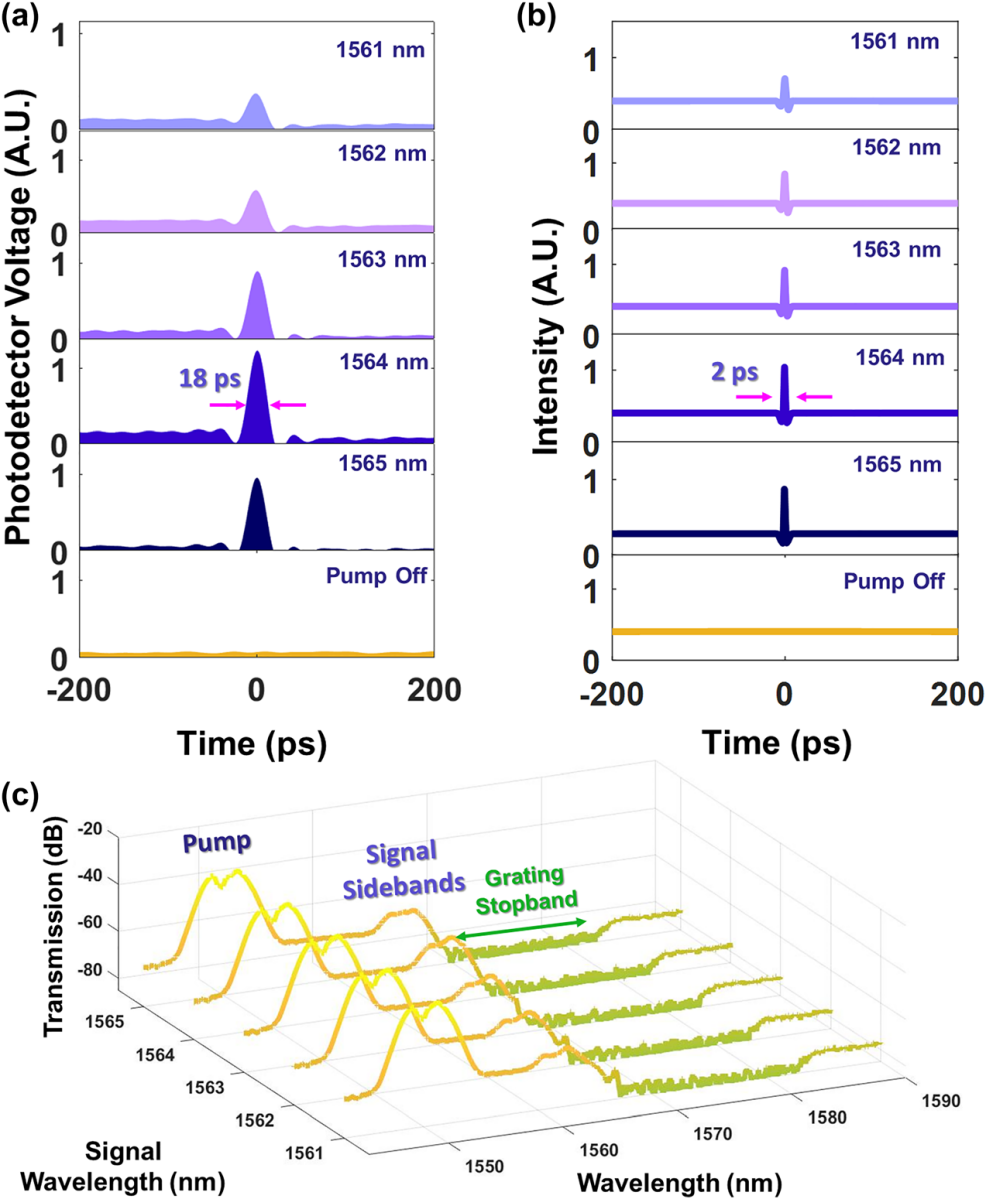
Temporal characterization of the signal waveform as its wavelength is varied (Configuration 1). The pump wavelength is fixed at 1550 nm and the signal wavelength varied between 1561–1565 nm. The stopband of the grating used in this experiment is centered at 1575 nm. (a) Measured and (b) numerically calculated signal waveform as a function of signal wavelength. (c) Measured output spectrum from the nonlinear grating as a function of the signal wavelength, where the sidebands are seen to develop around the signal. The input signal is filtered from the spectrum to clearly show the XPM-induced sidebands that formed.

Figure 3a shows the temporal characterization of a pump located at 1550 nm and signal (tuned from 1561–1565 nm), in Configuration 1. The signal is tuned within the region of high anomalous dispersion in the grating whereas the pump is located far from the grating stopband, and so experiences the background waveguide dispersion which is negligible on the length scale of the experiment. It is observed from Figure 3a that as the signal wavelength is tuned closer to the grating stopband (increased), the squeezing effect on the CW signal strengthens, as observed through the higher amplitude of the generated waveform. The stronger squeezing effect is consistent with the signal being tuned to a region of higher anomalous dispersion, facilitating greater temporal synchronization on both the red-shifted and blue-shifted components of the signal. We note further that in Configuration 1 the pump wavelength is fixed. Therefore, the stronger squeezing effect originates solely from the tuning of the signal wavelength. We note further that in both the experiments (Figure 3a) and numerical simulations (Figure 3b), troughs are seen to develop around the picosecond pulse that is generated. These arise as a result of energy conservation: The energy that heaps up into the pulse waveform needs to be acquired elsewhere, causing the energy in the immediate vicinity around the pulse to be reduced, developing troughs. This process is further accompanied by the appearance of spectral sidebands around the signal spectrum (Figure 3c), which also strengthen as the effect of the dispersion increases. The sidebands arise as a XPM of the signal with the pump. Numerical results in Figure 3b indicate that the picosecond pulses formed from the CW signal that temporally overlaps with the pump possesses a pulse width of 2 ps. Oscilloscope measurements shown in Figure 3a show a generated pulse full-width-half-maximum (FWHM) of 18 ps. The temporal resolution of the oscilloscope is limited due to its electrical bandwidth of 33 GHz.

**Figure 4:**
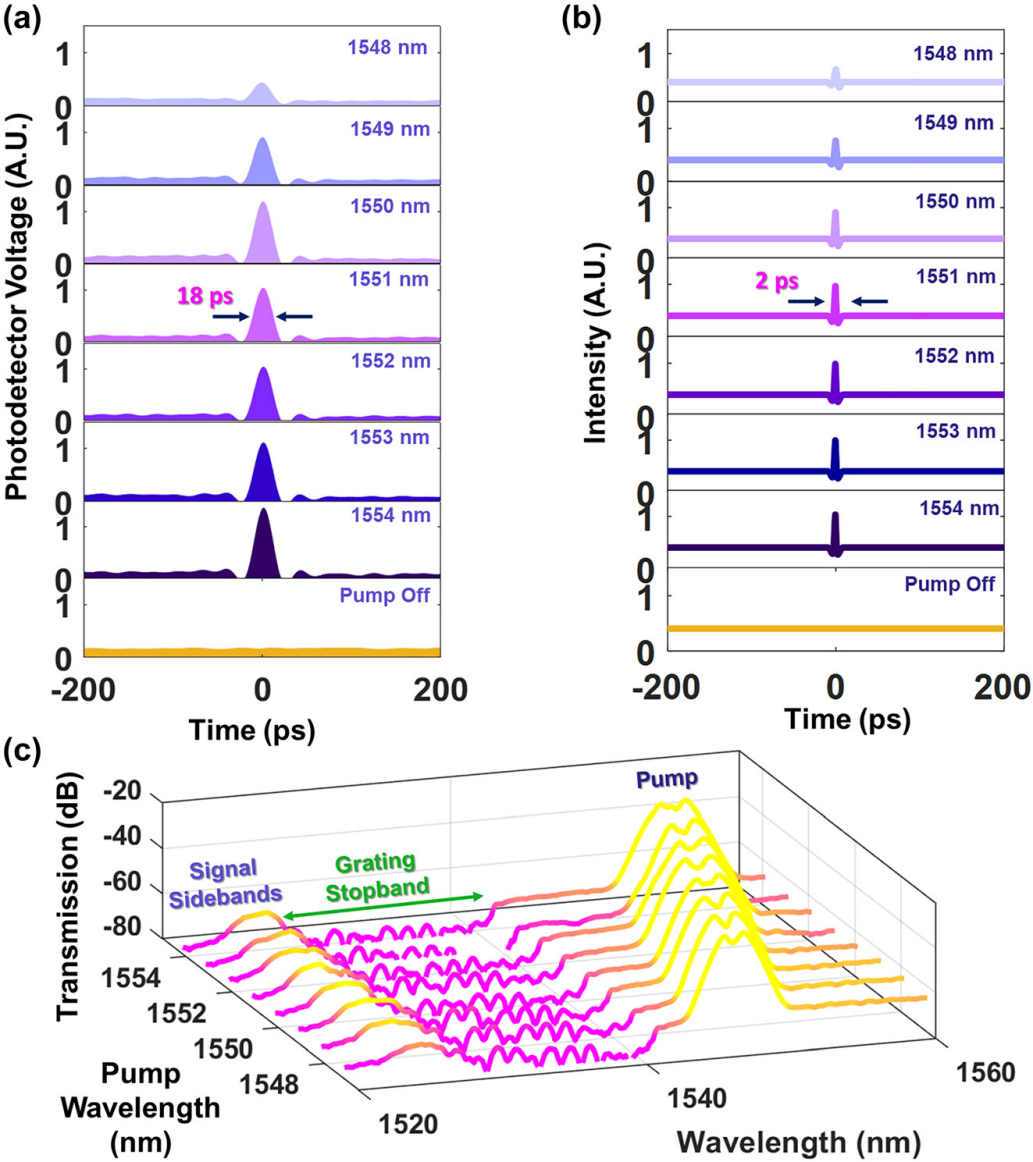
Temporal characterization of the signal waveform as a function of pump wavelength (Configuration 2). In this configuration, the signal wavelength is fixed at 1525 nm while the pump wavelength is varied between 1548–1554 nm. (a) The experimentally measured and (b) numerically calculated signal waveform as a function of pump wavelength. (c) Output spectrum showing the signal sidebands generated for the pump wavelength of 1547–1554 nm. The input signal is filtered from the spectrum to clearly show the XPM-induced sidebands that formed.

In keeping with the physical underpinnings of the squeezing effect, locating the pump on the opposite side of the grating stopband with the signal still located at the blue edge, should result in similar dynamics being observed. This is because the signal continues to experience anomalous dispersion induced by the grating, and XPM from the pump still occurs. In Configuration 2 the pump and the signal are both located close enough to the band edge for the grating dispersion to be substantial, and we again perform experiments to analyze the dynamics of the CW waveform. In this case, a grating with a stopband centered at 1535 nm is used (see Figure 2c). The CW signal wavelength is fixed at 1525 nm (blue edge of grating stopband, 3 dB bandwidth = 15 nm) and the pump is varied between 1548–1554 nm (red edge of grating stopband). The power of the pump and the signal are the same as in Configuration 1. Figure 4a shows the measured temporal profile of the CW waveform as a function of the pump wavelength. The region between 1548 and 1550 nm is where the pump pulses experience the greatest decrease in normal dispersion. This corresponds with an increase in the amplitude of the pulse which forms from the CW signal, a direct consequence of less severe temporal broadening (higher peak power) in the pump pulses. Beyond 1550 nm, the magnitude of normal dispersion experienced by the signal decreases much less rapidly, and as expected results in an almost steady state signal temporal profile. The development of troughs caused by the shift of energy from the immediate vicinity of the pulse to the peak region is observed here as well. Numerical calculations shown in Figure 4b show good agreement with the measurements, corroborating the experimentally observed increase in the amplitude of the pulse that develops, notably being most pronounced as the pump is turned from 1548 to 1550 nm. Spectral measurements shown in Figure 4c provide further confirmation, showing that the sidebands that develop around the signal via XPM for a pump wavelength of 1548 nm are the smallest. We note further that when the signal was placed at the red-edge of the stopband, no formation of picosecond pulses are observed in the CW signal, confirming the importance of the sign of the grating dispersion at the signal wavelength. In contrast, the sign of the dispersion at the pump wavelength is not critical for generation of the pulses. Numerical calculations shown in Figure 4b show that the pulses formed from the CW signal when temporally overlapping with the pump are expected to have an FWHM of 2 ps. It is observed that the signal FWHM in the experimental measurement is limited to 18 ps as a result of the limited oscilloscope temporal resolution. The quantitative trends observed in both experiment and numerical simulations agree remarkably well, including the troughs which develop around the generated pulse waveform.

**Figure 5:**
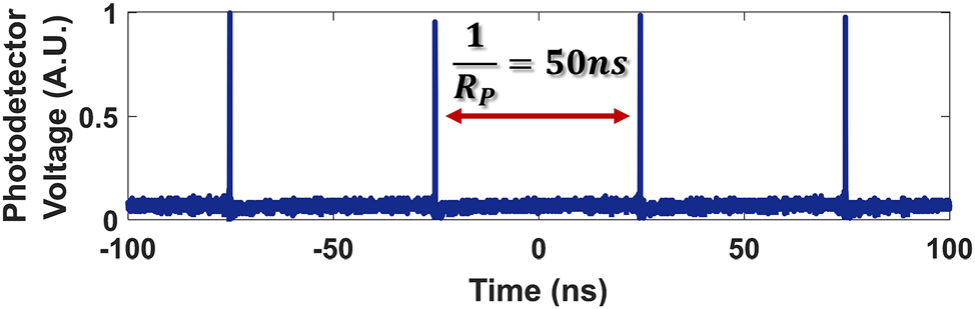
Oscilloscope measurements showing the pulse train generation from the CW signal occurring every 50 ns, corresponding to the repetition rate of the pump pulses.

In addition, our measurements indicate that the modification of the probe occurs only when the pump pulses are present. We perform oscilloscope measurements of the CW signal as a function of time for Configuration 2, over four repetitions of the pulsed laser (repetition rate = 20 MHz), as shown in Figure 5. Due to the salient role of the pump pulses in triggering the observed phenomena, the picosecond waveforms are generated only at instances when the pump pulses propagate (every 50 ns).

To further shed light on the dynamics associated with the generation of the picosecond pulses from the CW signal, we perform additional numerical calculations of the CW signal’s waveform’s evolution as a function of propagation distance in the 3 mm grating. The pulse propagation dynamics of the co-propagating pump and signal are governed by coupled nonlinear Schrödinger equations [25]:

(1) $$\frac{\partial A_{s}}{\partial z}+\frac{1}{\upsilon_{\text{g},s}}\frac{\partial A_{s}}{\partial t}+\frac{i\beta_{2,s}}{2}\frac{\partial^{2}A_{s}}{\partial t^{2}}+\frac{\alpha_{s}}{2}A_{s}=i\gamma_{s}\left({\left|A_{s}\right|}^{2}+2{\left|A_{p}\right|}^{2}\right)A_{s}$$

(2) $$\frac{\partial A_{p}}{\partial z}+\frac{1}{\upsilon_{\text{g},p}}\frac{\partial A_{p}}{\partial t}+\frac{i\beta_{2,p}}{2}\frac{\partial^{2}A_{p}}{\partial t^{2}}+\frac{\alpha_{p}}{2}A_{p}=i\gamma_{p}\left({\left|A_{p}\right|}^{2}+2{\left|A_{s}\right|}^{2}\right)A_{p}$$

**Figure 6:**
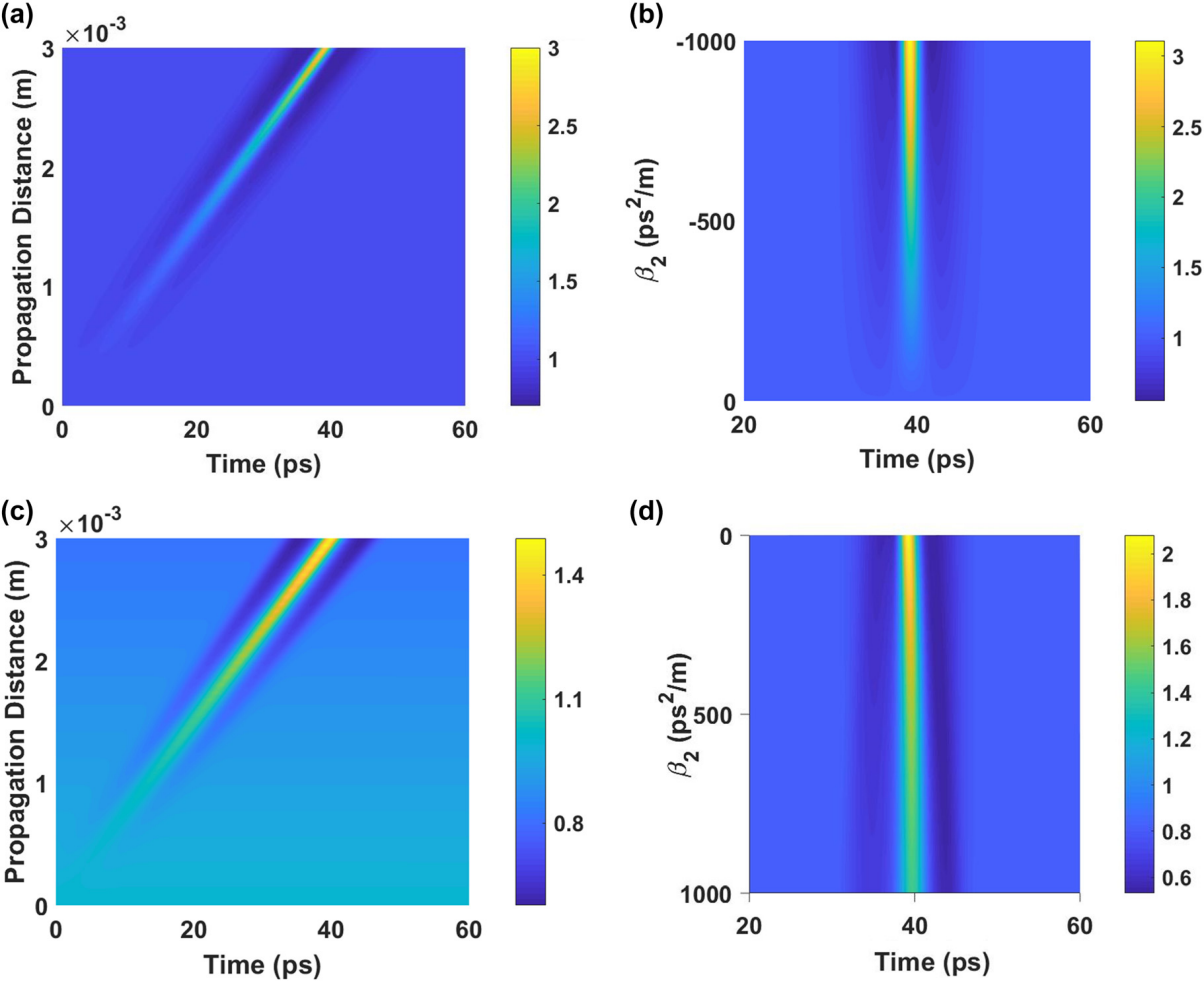
Numerical calculations of the temporal dynamics. (a) Signal waveform as a function of propagation distance for Configuration 1, and (c) similar but for Configuration 2. (b) Signal waveform versus probe dispersion, *β*_2_ in Configuration 1 (d) signal waveform versus pump dispersion, *β*_2_ in Configuration 2. Color bars denote probe power in milliwatts.

Here, subscripts *s* and *p* refer to the signal and pump. Note that the parameters used for the pump and signal will be at those corresponding to their wavelength location and respective electric-field amplitudes. *A*, *υ*_g_, *β*_2_ and *α* refer to the slowly varying amplitude, group velocity, GVD and loss coefficient, respectively. The effective nonlinear parameter, $\gamma ={\left(\frac{n_{\text{g}}}{n_{0}}\right)}^{2}\cdot \frac{2\pi n_{2}}{\lambda A_{\text{eff}}}$ where *n*_g_ is the group index, *n*_0_ is the core refractive index, *n*_2_ is the nonlinear refractive index of USRN, *λ* is the wavelength and *A*_eff_ is the effective area of the mode. Far from the grating stopband, *γ* approaches that of the waveguide without the cladding modulation (calculated *γ* = 500 W^−1^m^−1^). In the simulations, the wavelength-dependent grating parameters, experimental pump peak power (3.7 W) and signal average power (1 mW) are used. Figure 6a shows the evolution of the CW signal in Configuration 1 as it propagates through the grating. It shows that the CW signal attains an increasing modulation depth, developing a pulsed profile. As the CW signal propagates further into the grating, the interaction length increases, leading to a strengthening of the squeezing effect.

Figure 6b investigates the effect of increasing the magnitude of anomalous dispersion experienced by the probe in Configuration 1. Essentially, the signal experiences increasing magnitudes of anomalous dispersion as its wavelength is increased. As the dispersion magnitude increases, the squeezing effect conferred by the pulse increases as well. This is clearly observed in Figure 6b where the amplitude of the CW waveform’s modulation depth at the output of the grating, strengthens with the dispersion in the nonlinear grating, which in practice can be performed by varying the wavelength of the CW probe.

Figure 6c, which refers to Configuration 2, shows that the evolution of the signal waveform is similar to that in Configuration 1. However, it is observed from Figure 6d that the strength of the squeezing effect decreases as the pump is tuned closer to the edge of the grating since decreasing its detuning results in stronger (normal) dispersion. In turn this results in greater temporal broadening of the pump, hence leading to lower pump peak power, and a concomitant reduction in the XPM imparted to the CW waveform.

The dispersion length is given by $L_{\text{D}}=\frac{{T_{0}}^{2}}{\left|\beta_{2}\right|}$ where *T*_0_ is close to the pulse’s FWHM [25]. Varying the dispersion between 200 ps^2^/m to 1000 ps^2^/m as the detuning from the center of the stopband varies from 10 to 8 nm, implies dispersion lengths between 8 and 40 mm. Based on the dispersion lengths relative to the physical length of the grating, it may be concluded that when the detuning with the stopband is large, the dispersion length is sufficiently long relative to the 3 mm grating length to have negligible impact on the temporal broadening dynamics of the pump pulses, whereas close to the stopband, the dispersive length of 8 mm implies a noticeable impact on the pulse propagation dynamics.

## 3 Discussion and conclusion

We note that microring resonators may also be used to generate optical pulses from CW light. Frequency combs generated by parametric oscillation in microring resonators do not automatically confer coherence to the comb lines. It has been shown in Ref. [26] that this issue may be overcome by controlling the phase properties of each comb line using a spatial light modulator (pulse shaper), such that an input CW signal may form pulses. This approach however requires complex off-chip systems which might not be compatible with the goal of integrated systems [26]. Soliton states in microring resonators overcome this hurdle, with the pulse width of the derivative pulses being dependent on the free-spectral range of the ring [27]. Compared to our approach which is a non-resonant mechanism, solitons in microring resonators are susceptible to thermal effects. Soliton states have been reported in Ref. [27] to be susceptible to a thermally induced resonance shift, which is a slower effect than the instantaneous Kerr induced resonance shift. Therefore, the added requirement of being in thermal equilibrium for the soliton state to be achieved makes it less straightforward to implement compared to our approach. In nonlinear ring resonators, the dynamic range for wavelength tuning of the generated pulse is limited. In addition, careful control of the pumping conditions is required to achieve the single soliton state, as opposed to having multiple co-propagating solitons within the resonator.

The approach for generating pulses from CW light demonstrated in our work provides a more easily implemented alternative with additional design knobs. In our configuration, the pulse generated from the CW input signal can be tailored across a wide wavelength range. Since the pulse generation mechanism relies on the combined effects of XPM, and the high dispersion induced by the grating whose center wavelength can be easily controlled by varying the pitch, picosecond pulse generation from CW signals at a multitude of wavelengths may be achieved even in the absence of anomalous dispersion in the background waveguide or material platform used. In other words, as long as a CW signal may be positioned on the blue-edge of the grating stopband (the stopband of which can be tailored using its pitch), the generation of a picosecond pulse train can be achieved.

Compared to nonlinear ring resonators, our approach allows very low power CW signals to undergo picosecond pulse generation. Ref. [26] for example, requires a CW power between 66 mW and 1.4 W. Conversely, we demonstrate the generation of picosecond pulses with a low power of 1 mW, 2–3 orders of magnitude smaller than that reported in Ref. [26].

Within the context of on-chip temporal compression as another approach for generating short pulses, most approaches thus far have relied on the nonlinear effects self-induced by a pulse. Soliton-effect compression has been demonstrated in a variety of CMOS-compatible media including silicon [24, 28], Hydex glass [29], USRN [17, 30] and silicon nitride [31, 32] have shown good performance. Compression in waveguides leverage the initial temporal narrowing experienced by a very high order soliton [25], while photonic crystal waveguides leverage both the slow light effect and augmented anomalous dispersion on the band edge, induced by the anti-crossing between the index guided and gap guided modes in the photonic crystal [33]. More recently, high spectro-temporal compression was demonstrated in a silicon-based two-stage compressor system which decouples the nonlinear and dispersive effects, resulting in 11× temporal compression, a 9.4× enhancement in the output peak power, and 3× spectral compression of optical pulses [20]. In these schemes, self-phase modulation was central to the compression process, indicating that the nonlinear phase acquisition scales linearly with the input peak power of the pulse to be compressed. Consequently, the extent of compression hinges on the power of the optical waveform to be compressed. It is therefore challenging to derive picosecond pulses from weak power signals using this scheme. CW light poses a further obstacle, in that its DC nature implies that it does not benefit from a peak power increase that relates inversely with its associated pulse width.

Here, we overcome this intrinsic hurdle, by demonstrating pulse train generation of very low power optical signals (1 mW) to 18 ps, and more importantly, optical waveforms which are CW in nature. Since the underlying mechanism by which the picosecond pulses are generated relies on (i) XPM from a strong pump for new frequency generation, imparting both blue- (trailing edge) and red-shifted (leading edge) and (ii) anomalous dispersion to temporally retard (accelerate) red- (blue-) shifted frequencies generated via XPM, weak powered, CW light can be efficiently manipulated. The combined effect is to squeeze the light on two ends of the CW probe temporally overlapping with the pump inwards, drawing energy from the light around its immediate vicinity. In both experiments and numerical simulations, energy conservation is manifested as the troughs that are seen to develop around the squeezed CW probe. We note further that the grating dispersion of the probe provides a knob with which to vary the profile and intensity of the generated picosecond pulses. In the future, the possibility of tailoring the repetition rate of the generated picosecond pulses could be studied by adopting integrated approaches for temporal interleaving of the signals [34, 35]. This work introduces a new approach in which temporal waveforms may be manipulated towards possible applications in tunable pulse train generation, wave-shaping and optical time division multiplexed high-speed data transmission.

## Supplementary Material

Supplementary Material
