# Supplementary material for: Picosecond pulse generation from continuous-wave light in an integrated nonlinear Bragg grating
Source: Nanophotonics. 2022 Mar 18;11(10):2319–28. doi: 10.1515/nanoph-2022-0026 (PMC11636417; doi:10.1515/nanoph-2022-0026)
Supplement: Supplementary file 1 — Supplementary Material [file j_nanoph-2022-0026_suppl.docx]

**Supplementary Information**

**Picosecond pulse generation from continuous-wave light in an integrated nonlinear Bragg grating**

Ju Won Choi^1+^, Byoung-Uk Sohn^1+^, Ezgi Sahin^1^, George F. R. Chen^1^, Doris K. T. Ng^2^, Benjamin J. Eggleton^3,4^, C. Martijn de Sterke^3,4^ and Dawn T. H. Tan^1,2,*^

^1^Photonics Devices and System Group, Singapore University of Technology and Design, 8 Somapah Rd, Singapore 487372, Singapore

^2^Institute of Microelectronics, A*STAR, 2 Fusionopolis Way, #08-02, Innovis Tower, Singapore 138634, Singapore

^3^Institute of Photonics and Optical Science, School of Physics, The University of Sydney, Sydney, New South Wales 2006, Australia

^4^The University of Sydney Nano Institute (Sydney Nano), The University of Sydney, Sydney, New South Wales 2006, Australia

+These authors contributed equally to this work.

*Corresponding author: dawn_tan@sutd.edu.sg

**S1. Comparison of measured signal waveforms with additional configurations where signal is normal dispersion.**

We perform the experiments while situating the signal on the red-side of the grating, where the dispersion is normal. It may be seen in the following figures that in this case, the signal does not undergo the pulse train generation and retains its continuous wave nature. From this standpoint, we may deduce that formation of the pulses from the CW signal relies on the presence of anomalous dispersion from the grating.

In addition, Figures 3 and 4 in the main manuscript show the measured oscilloscope trace when the pump is off. In this scenario, no compression is observed.


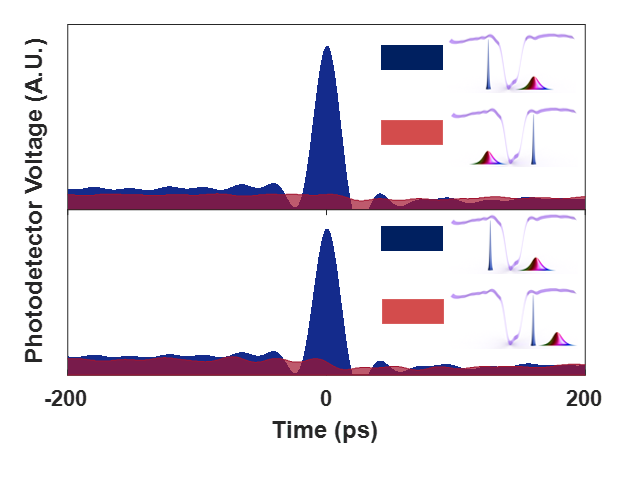


**Figure S1**. The measured oscilloscope traces for two additional configurations as shown in the legend, plotted on the same scale as the measured oscilloscope trace for Configuration 2.

**S2. Comparison of input and output spectra**

Figure S2 shows the input pump and continuous-wave signal (black line) prior to coupling into the sample for the experiments plotted in Figure 4 of the main text (Configuration 2, signal wavelength=1561 nm). It is clearly shown that signal sidebands are created, and the pump is broadened after passing through the sample (red line). The linear transmission spectrum shown in blue reveals a bandgap of 1567.5nm – 1582.5nm. The output spectrum of signal when the pump is off is shown as the dashed magenta line. In this case, no sidebands develop around the signal.

**Figure S2.** The output spectra before (black line) and after (red line) the device. The dashed magenta line shows the output spectrum of the signal when the pump is off.
